# Predicting altcoin returns using social media

**DOI:** 10.1371/journal.pone.0208119

**Published:** 2018-12-04

**Authors:** Lars Steinert, Christian Herff

**Affiliations:** Cognitive Systems Lab, University of Bremen, Bremen, Germany; Institut Català de Paleoecologia Humana i Evolució Social (IPHES), SPAIN

## Abstract

Cryptocurrencies have recently received large media interest. Especially the great fluctuations in price have attracted such attention. Behavioral sciences and related scientific literature provide evidence that there is a close relationship between social media and price fluctuations of cryptocurrencies. This particularly applies to smaller currencies, which can be substantially influenced by references on Twitter. Although these so-called “altcoins” often have smaller trading volumes they sometimes attract large attention on social media. Here, we show that fluctuations in altcoins can be predicted from social media. In order to do this, we collected a dataset containing prices and the social media activity of 181 altcoins in the form of 426,520 tweets over a timeframe of 71 days. The containing public mood was then estimated using sentiment analysis. To predict altcoin returns, we carried out linear regression analyses based on 45 days of data. We showed that short-term returns can be predicted from activity and sentiments on Twitter.

## Introduction

**Fig 1:**
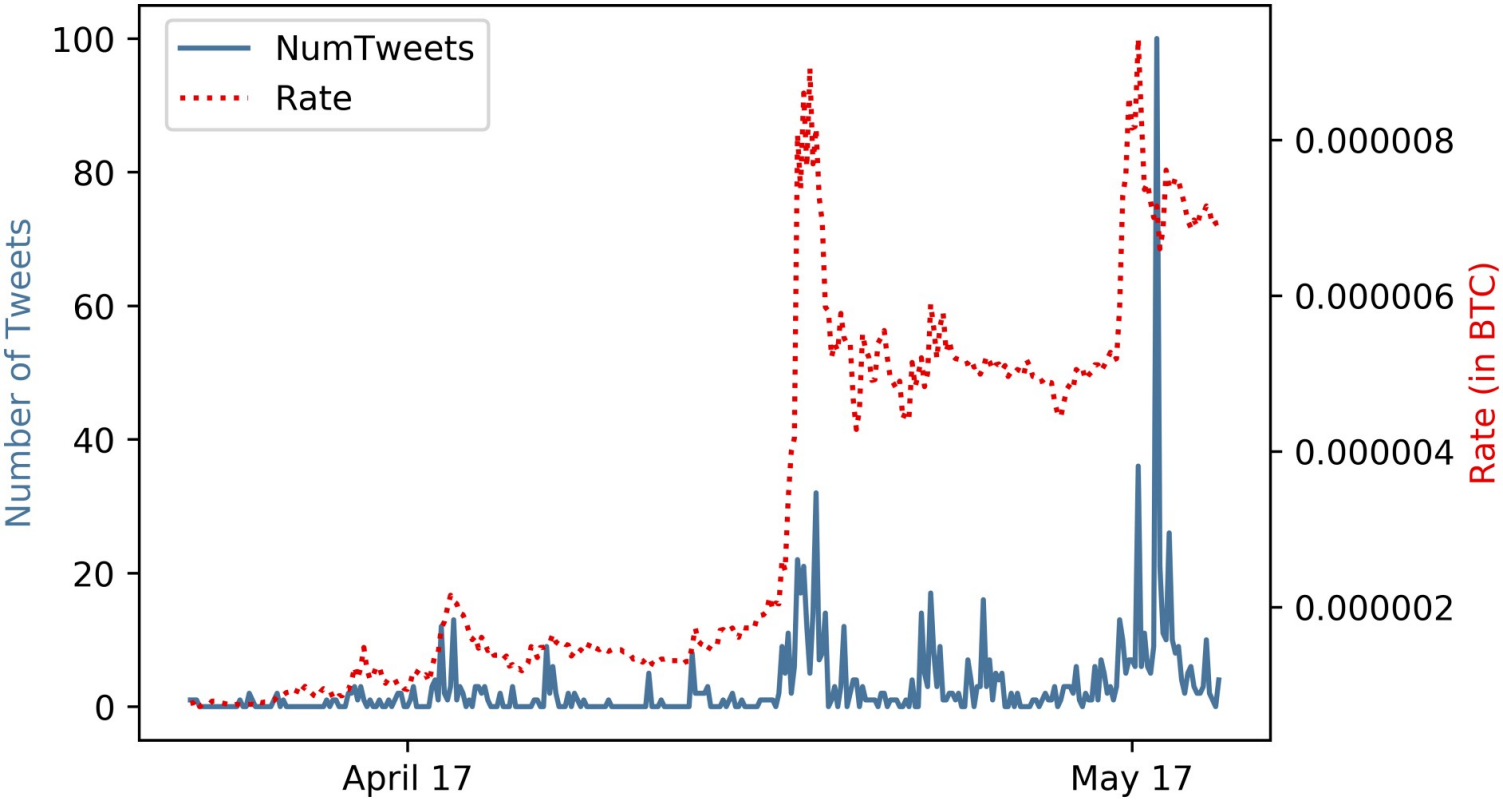
Number of tweets (solid) and rate (dashed) of PinkCoin over a time period of 45 days. A moderate Pearson correlation (*r* = 0.438) between the price of PinkCoin and the number of tweets referring to the cryptocurrency is visible.

Until a few years ago, cryptocurrencies hardly received any public attention. Only few people had heard about Bitcoin, if at all. This changed with the emergence of the illicit goods marketplace Silk Road and the public’s growing interest in the so-called “darknet”. It seemed that all of a sudden, Bitcoins were perceived as the currency for illegal purposes. Today, cryptocurrencies increasingly assert a place in the awareness of society and the public. This is accompanied by an increasing research interest. Thus, a rising number of studies have placed their focus on Bitcoins and cryptocurrencies in general. Two questions that many of these studies address are: How are returns of cryptocurrencies determined and is it possible to predict them? As for traditional commodities, there is reasonable evidence to suggest that cryptocurrency returns are somehow connected to public perception. Cryptocurrencies tend to be highly volatile which makes them sensitive to external factors. This attracts groups who are trying to take advantage of this circumstance by applying typical “pump and dump” schemes [1]. In such a scheme, chosen cryptocurrencies are purchased in small, inconspicuous amounts in a certain time range. In total, these purchases then add up to a substantial amount. Secondly, these cryptocurrencies are then heavily promoted via social media, which leads to purchases by other parties. The corresponding price increases provides the persons initiating the scheme with very high returns. This particularly applies to smaller cryptocurrencies, so-called “altcoins”. Altcoins are easier to influence and are thus of special interest in this study. Although there is no existing conformity regarding the term “altcoin” in scientific literature, we agree with the majority of authors who classify every alternative to Bitcoin as an altcoin [2, 3]. Another factor that suggests a connection between cryptocurrency returns and social media is based upon theories of behavioral sciences. Social media platforms such as Twitter can be seen as a collective indicator of thoughts and ideas but also of public mood [4]. Positive mood states lead to more optimism towards investment decisions [5]. Thus, social media might be a good prediction indicator for investments in cryptocurrencies, of which the extents are reflected in their prices. While there are all sorts of social media information available, we believe that it is especially the short-term variability of Twitter that makes the platform a very suitable indicator for short-term predictions. Furthermore, unlike services such as Google Trends, Twitter does not solely provide information about activity but also about the associated mood. Fig 1 illustrates the price and the social media activity which is represented by the number of tweets for the cryptocurrency PinkCoin. At first glance, one would suspect a connection.

In the scope of this work, we wanted to pay closer attention to these indications. Over a time range of 71 days, we collected a dataset covering 181 altcoins. In terms of the scientific literature, this is an unparalleled achievement. Based upon this data, we ran linear regression analyses in order to create short-term prediction models. The aim of these models was to analyze whether it is possible to predict altcoin returns based upon social media activity. The activity was measured by the number of tweets that referred to a certain altcoin and the sentiment that they contained. We therefore hypothesized that it is possible to predict the return of an altcoin based upon the number of tweets that are referring to it and the sentiment contained in said tweets. The scientific interest in cryptocurrencies is relatively new. However, there is some related work in existence. Some papers deal with Bitcoin users [6–8], Bitcoin in general [2, 9–13] or the market dynamics of cryptocurrencies [14–16]. Fewer papers have have analyzed the relationship between cryptocurrency prices or returns and social media activity. While some authors have based their analysis on online forums [17, 18], Google Trends and Wikipedia [19] or social media platforms like Reddit [20], others have used data provided by Twitter [21]. Authors such as Garcia and Schweitzer [22] have even created a framework to bundle several social and economic signals in order to predict the return rates of Bitcoins. Kristoufek [23] has investigated the most frequently claimed drivers of the Bitcoin price. Several authors argue that Bitcoins, consequently cryptocurrencies in general, have mainly been addressed in a speculative frame [7, 24, 25]. Therefore, one could say it may be justified or even more appropriate to treat cryptocurrencies much like stocks rather than fiat currency. Works such as [26, 27] have concentrated on the prediction of Dow Jones Industrial Average (DJIA) and various stock market indices respectively. Both studies have used tweets including certain expressions of mood. While Bollen *et al*. [26] have been able to demonstrate that some mood dimensions have a high causality towards the DJIA closing values, Zhang *et al*. [27] have found negative moderate correlation, among other things, between the ratio of tweets that contain signals of hope and all considered indices, namely Dow Jones, NASDAQ and S&P 500, with a one-day time lag. While we were assessing the above-mentioned literature, we came across a few things that stood out. No cryptocurrency-related paper, except for one, has considered other cryptocurrencies than Bitcoin in their analyses. This might be due to the superior interest in Bitcoin, or there may even be a more trivial reason: many people may not even know of the existence of other cryptocurrencies. Secondly, all cryptocurrency-related papers have only focused on daily prices. Cryptocurrencies tend to be extremely volatile. Therefore, considering intraday prices would allow a more granular, detailed picture. It is also notable that among all papers the sentiment analysis approaches vary. Some authors [4, 21] have assigned tweets a class (positive, neutral, negative) and have used the number of tweets of each for their analysis. Other authors such as Kim *et al*. [17] have applied a valence-based approach which also considered the strength of the sentiment. There are many more studies in existence that have attempted to predict stock market prices using different factors. Especially the prediction using public sentiments seems to be of superordinate interest. For example, some authors have also used sentiments on Twitter [28–33] whereas others have used sentiments from stock message boards [34, 35]. However, the scientific interest in cryptocurrencies is relatively new and therefore, there are only a small number of studies. This is understandable because cryptocurrencies as we know them today started to exist with the appearance of Bitcoin, which is less than ten years ago [36]. Based on these grounds, we aimed to expand the field by increasing the quantity of cryptocurrencies included in our analyses. In order to do this, we collected data of the 181 largest altcoins. We also paid attention to the emotional value of tweets pertaining to cryptocurrencies. We showed how Twitter activity and sentiment could be used for some currencies to predict altcoin returns. This is the first time that small cryptocurrencies have been rigorously investigated for their prediction potential based on Twitter. To further support this growing field, we share the collected dataset with the scientific community in the Supporting Information (S1 File).

## Materials and methods

For this work, we collected altcoin prices and social media activity of 181 altcoins over a period of 71 days in total. Both, prices and social media activities were updated every three hours to also allow for short-term analysis. These datasets are not continuous but split into two time periods. The first period starts on March 21 and ends on May 5, 2017 and thus covers 45 days. The data collected in this range represented the training set. The second period covers 26 days and starts on May 9 and ends on June 4, 2017. The data obtained in this range functioned as the test set. This set was used to evaluate the models based on the training set. The ratio of these sets is around 1.73:1. In total, our datasets include prices for 181 different altcoins and 426,520 tweets referring to them. All datasets were collected in a legitimate manner, fully complying with the terms of service of the sources in use. The sources of our data will be explained in the next sections.

### Twitter data

To collect tweets referring to the altcoins we used the Twitter Search API [37]. Using this, one can directly crawl Twitter data including usernames, hashtags, tweets, the associated timestamp of tweeting and the number of retweets. In our case, a query referred to a certain altcoin. This was done by using the official currency codes. These are for example *$ETH* for Ethereum and *$LTC* for Litecoin and so on. We collected tweets that were retweeted just as we collected tweets that point to several altcoins. This was to ensure, that the picture that was presented to the twitter community, gets reflected in our data corpus. Due to restrictions placed by Twitter it was only possible to gather a random sample of up to 100 tweets per query resulting in updated social media activity every three hours for each altcoin. Queries were processed at three-hour intervals and contained up to 100 tweets that had been posted since the last query. For each query, we saved all tweets and the total number of tweets.

### Sentiment analysis

**Fig 2:**
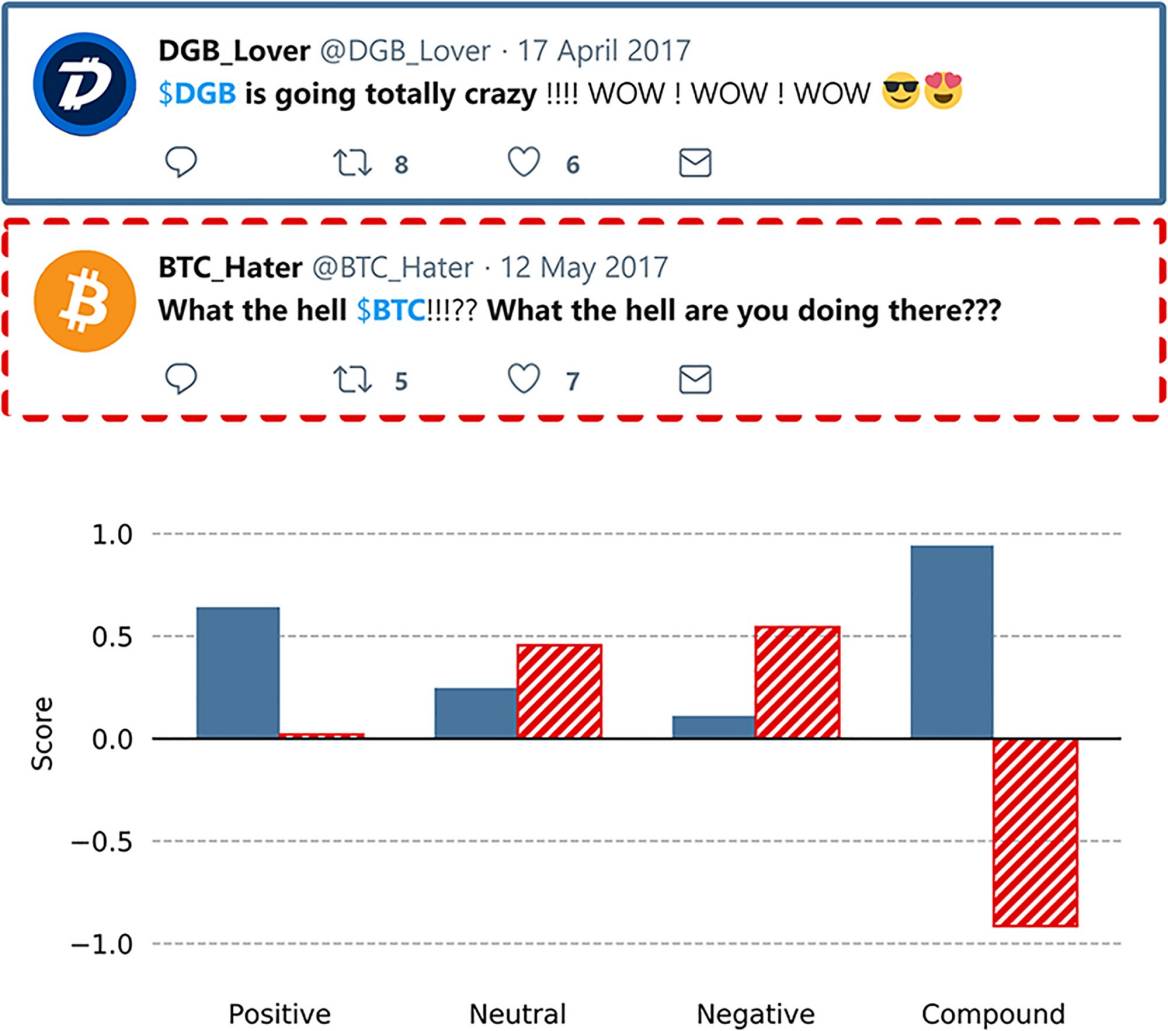
Visualization of VADER scores for two artificially generated example tweets. The tweet highlighted in blue contains different positive expressions. In contrast, the red dotted tweet contains negatively connoted language. Accordingly, both tweets differ widely in terms of the four computed VADER scores.

In order to extract the sentiment expressed in a tweet, we applied VADER, a valence-based sentiment analysis model that especially addresses the analysis of social media text such as tweets [38]. Tweets often contain language variation and show the frequent usage of emoticons, abbreviations or slang [39]. These special characteristics lead to particular requirements when it comes to sentiment analysis in order to avoid information loss. VADER meet these demands by relying on lexicons that contain Western-style emoticons and sentiment-related acronyms [38]. However, there are several other sentiment analysis approaches in existence that will lead to a similar outcome. When applying VADER for sentiment analysis, four different scores are computed: positive, neutral, negative and compound. The positive, neutral and negative scores are portions or segments of text that are matched to their respective group. These segments, when added to each other, should total a sum of 1. The compound score represents the sum of each’s word valence in the lexicon. This sum is then normalized to a score between -1 (extremely negative) and +1 (extremely positive). Fig 2 visualizes the scores that were calculated using VADER on two artificially generated example tweets. The tweet marked in blue contains several positive expressions such as “WOW” and happy emoticons. Consequently, the tweet has a high positive score and a highly positive compound score. The tweet highlighted in red dots expresses the opposite by using the negatively connoted expression “What the hell are you doing????”. This leads to a high negative score and a highly negative compound score. The above-mentioned scores were calculated for every single tweet. These tweets were grouped according to their timestamp by calculating the mean of each score. This allowed us to assign these scores to the referred altcoin and subsequently use them as features for the prediction models.

### Currency data

The finance-related dataset was obtained via CryptoCoinCharts API which is offered by the online platform CryptoCoinCharts [40]. Said platform provides an overall view of the cryptocurrency market. Alongside information about different cryptocurrency exchanges, it also includes information pertaining to certain cryptocurrencies themselves, such as exchange rates, trading volume, the current supply and the official currency codes. Accordingly, we used the CryptoCoinCharts API for gathering the market data. For our analysis, we considered all altcoins for which CryptoCoinCharts provided data over the complete time frame. This amounted to 181 altcoins in total. Because of its superior market position, it is common practice in cryptocurrency exchanges to define prices in *BTC*. This also applies to CryptoCoinCharts. Price information is updated in the same three-hour intervals as social media activity of the same cryptocurrency. By synchronizing the timing of obtaining the financial and social media data, we ensured that there is no information leakage. Table 1 illustrates a summary statistics of our dataset.

**Table 1:** Summary statistics. This table describes our training set. The presented measures were calculated based on 60,273 samples covering 181 altcoins in total and provide an overview over the data that was analyzed in this study. The volume and rate are defined in BTC.

Variable	Mean	S.D.	Min	Max
NumTweets	3.635	12.243	0.000	100
Positive	0.032	0.068	0.000	0.700
Neutral	0.354	0.443	0.000	1.000
Negative	0.008	0.032	0.000	0.727
Compound	0.058	0.161	-0.922	0.956
Volume	637.150	3736.074	0.000	97574.006
Rate	0.006	0.057	0.000	0.912

### Data preprocessing

It was the case that some altcoins for which we gathered market data had none or almost no social media activity on Twitter referring to them. In order to create reliable predictive models, however, it is essential to have a substantial amount of data. For this reason, it was not expedient to consider those altcoins in our analyses that did not meet these requirements. Therefore, we only included those altcoins that were referred to on Twitter on at least 10% of all days in our training set. By choosing a relatively low threshold we wanted to ensure that we consider as many altcoins as possible in our analyses. This approach led to a total number of 131 altcoins that we took into consideration in the subsequent steps.

### Prediction approach

In order to create prediction models, we applied ordinary least squares linear regression analyses on our training set. The number of tweets and all scores that were calculated using VADER were used as input features. This resulted in a total number of 333 samples with 5 features, namely the number of tweets of the last three hours and the means of the four VADER scores of these tweets, for each currency. Instead of considering the rates as the response variable, we used the altcoin returns. In contrast to rates, returns are assumed to be less autocorrelated and allow for direct investment strategy adjustment. Therefore, using returns instead of rates reduced the risk of receiving results due to autocorrelated data. The altcoins returns were calculated for a time horizon of *t* = [1, 8]. As samples were gathered at three-hour intervals, one time lag corresponds to a three hour gap. Therefore, the following is valid:

$$\begin{array}{c} return_{t}=price_{t}-price_{0} \end{array}$$ (1)

Accordingly, we calculated the returns for up to 24 hours in the future. The described approach was applied to all 131 altcoins. However, we did not analyze and evaluate every model in a detailed manner. Instead, we focused on a small set of altcoins that indicated the highest connection between the input features and the returns. This was measured using the mean *R*^2^ value for the complete time period of the training set. The *R*^2^ value, or coefficient of determination, represents the explained proportion of variation of the dependent variable, here returns, by the independent variables, activity and sentiment. Hence, we set our focus on those five altcoins that showed the highest mean *R*^2^ value for all time lags to find those model with an good average short-term predictability. These models were applied to new, unseen data from our test set. In order to take the number of statistical tests into account and thus to reduce the risk of a type I error, we applied Bonferroni correction.

## Results

**Fig 3:**
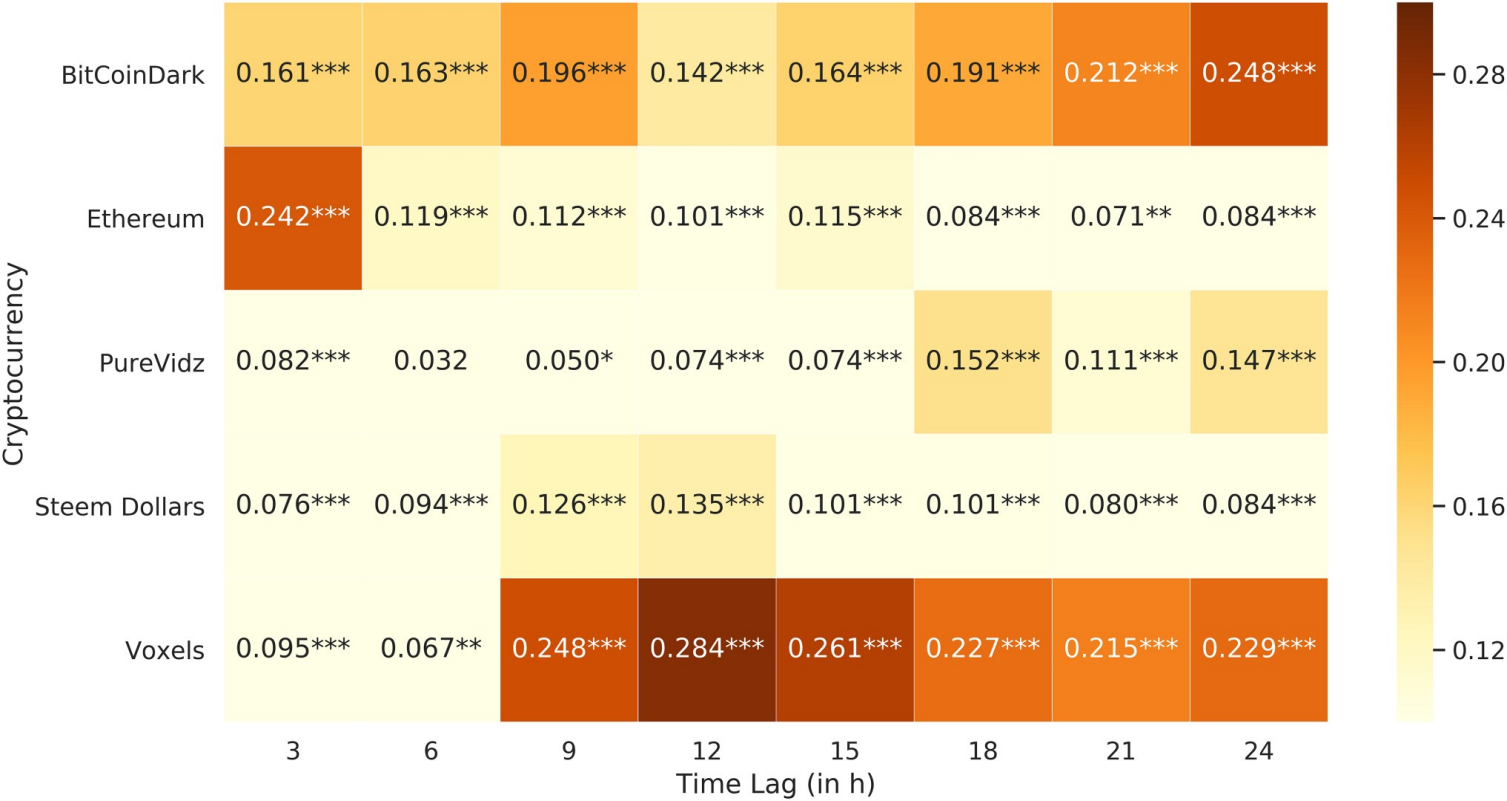
*R*^2^ values of the models based on the training set. High *R*^2^ values were encountered for some lags. Levels of significance (Bonferroni-corrected): *** p-value<0.001, ** p-value<0.01, * p-value<0.05.

Applying linear regression analyses to our gathered training data led to a wide range of results. Whilst some altcoin returns did not seem to respond to changes in social media activity and public mood on Twitter at all, some clearly did. Fig 3 visualizes the *R*^2^ values of the five selected altcoins for all time lags. The selection based on the highest mean *R*^2^ value did not provide a homogeneous picture. Instead, this approach selected currencies with a large variety of social media activity, trading volumes and prices. According to its market capitalization, Ethereum is the biggest altcoin [41]. Ethereum correspondingly generated much interest on Twitter (Mean NumTweets: 61.4) and had a high trading volume (Mean Volume: 29,825.8 *BTC*) and rate (Mean Rate: 0.0437 *BTC*). This cryptocurrency had particularly high *R*^2^ values in the first time lag (*R*^2^ = 0.242), or in other words, after three hours. This means that this model was especially suitable for explaining very short-term variations in returns. In contrast, BitCoinDark, Voxels and PureVidz are rather small altcoins. BitCoinDark generated little social media interest (Mean NumTweets: 1.05) and had a rather low trading volume (Mean Volume: 119.7 *BTC*). This also applied to Voxels (Mean NumTweets: 1.55; Mean Volume: 107.19 *BTC*), Steem Dollars (Mean NumTweets: 0.19; Mean Volume: 123.10 *BTC*) and PureVidz (Mean NumTweets: 1.11; Mean Volume: 0.89 *BTC*). The latter presented a particularly low level of trading reflected in the mean volume. Voxels showed a real increase in *R*^2^ values after 9 hours and reached the highest value of all models after 12 hours. Overall, BitCoinDark provided relatively high *R*^2^ values for all time lags whereas Ethereum really provided a peak value in the first lag.

**Fig 4:**
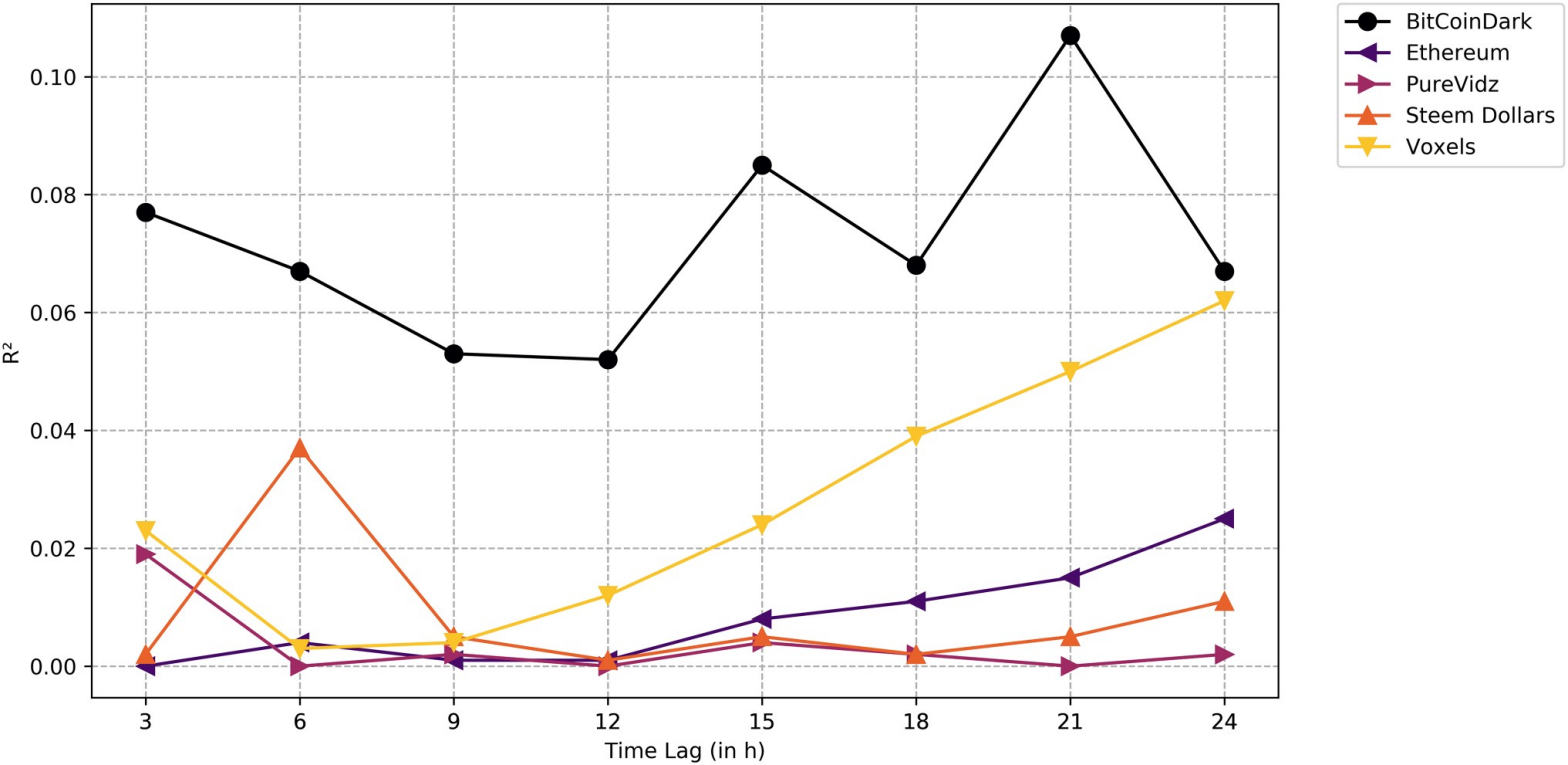
*R*^2^ values of the models based on the test set. Overall, all *R*^2^ values dropped. Statistical significance was found for 16 out of 40 predictions.

When applying these models to new data, the picture changed referred to the mean *R*^2^ value for all considered predictions. This was not surprising as the model was based on the training data. Accordingly, the *R*^2^ values for all altcoins on the test set were lower than on the training set as can be seen in Fig 4. However, not only did the mean *R*^2^ values for all models change but also the time lags for which our models best fit. Especially Ethereum stood out. On the training set, the model was particularly suitable for explaining the variation in the first time lag. Evaluated on new data, the model provided a significant connection in the last time lag. In the opposite direction this was also valid for PureVidz. As on the training set, *R*^2^ values for Voxels were higher in the later time lags than in the earlier ones. BitCoinDark still showed the highest mean *R*^2^ value over all time lags. After applying a Bonferroni correction, we found statistical significance for 16 out of 40 predictions. To ensure that a significant effect between our predictors and the returns existed, we also conducted a joint correlation analysis based on both the training and test data with fixed effect for coin type and found a significant fit of the model (f = 2.14e-249, p<0.001) with significant effects of number of tweets (p<0.001) and neutral sentiment (p<0.001).

## Discussion

Twitter has been the center of attention of many different studies related to the prediction of Bitcoin [21] and stock market indicators [26–30, 32] or even election results [42, 43]. The focus of this study was to examine whether the platform provides information that can be used to determine altcoin returns. This information was measured in five different dimensions: the extent of Twitter activity quantified as the number of tweets and the containing public mood measured as the average positive, neutral, negative and compound score. The aim of this work was to answer the question of whether altcoin returns can be predicted based upon this given information. We used our training set, which includes 45 days of altcoin prices and the above stated information for creating linear regression models. Instead of using the prices themselves, we calculated the returns at three-hour intervals for 24 hours. Only those altcoins, which had social media activity referring to them on at least 10% of the days in the observation period, were taken into further consideration. We created linear regression models for all considered altcoins and focused on those five which were most suitable in explaining the variance in returns measured as the mean *R*^2^ value over all time lags. These models were then evaluated using unseen test data covering 26 days after the training period. To ensure that there is not autocorrelation we set a pause of four days in between the training and the test set. We found statistically significant results for every considered altcoin for at least one time lag. In summary, this led to statistically significant results for 16 out of 40 predictions. The highest *R*^2^ values on our test set were reached by BitCoinDark (*R*^2^ = 0.107) and Voxels (*R*^2^ = 0.062). As far as we know, there is no other study in existence that has applied a similar approach to ours in order to predict cryptocurrency returns. Therefore, reference values are not easily available. As mentioned before, several authors argue for treating cryptocurrencies as an object of speculation. For this reason, it is reasonable to also allow the use of the results from studies relating to stock market prediction. Using sentiments from stock message boards to predict changes in stock prices, Das and Chen received an *R*^2^ value of 0.0027 [34]. Antweiler and Frank reported *R*^2^ values of 0.049 when predicting stock market prices also based on stock message boards [35]. Tetlock *et al*. used fractions of negative words in firm specific news stories to predict stock returns on the next day and found *R*^2^ values of up to 0.0024 [44]. Considering these examples, the found *R*^2^ values of up to 0.107 for the returns of BitCoinDark after 21 hours compare very well. Even the lowest statistically significant *R*^2^ value of 0.025 for Ethereum after 24 hours compares favourable to most of the existing literature. Although, again, the field of application and the underlying data is not the same, these studies provide an approximate scale for our results, which compare favorably. This pointed in the same direction as some of the related work. Garcia and Schweitzer [22] have shown that emotional valence and opinion polarization in tweets can be suitable predictors for Bitcoin returns. However, our models did not display a unified picture regarding the time span between changes in social media activity and public mood on Twitter and corresponding changes in altcoin returns. Whereas some altcoins seemed to respond quickly after three hours such as Steem Dollars, others such as Ethereum showed statistically significant response after 24 hours. It is noteworthy that the second biggest altcoin, according to its market capitalization, Ethereum, responded to changes in activity and the public mood on Twitter. This result correlates with the results provided by Kim *et al*. [17]. The authors found a significant relationship between positive and negative replies in an Ethereum-related online forum and the cryptocurrency’s price in 3 to 13 days in the future. Based on our findings, we can definitely state that the hypothesis, which postulates that there is no significant influence of the social media activity and the containing sentiment on future altcoin returns, can been rejected. Due to this circumstance, altcoin returns can be predicted to some extent by using the information provided by Twitter. Considering these results in a superordinate context, they do agree with the existing literature. Several studies are dedicated to the prediction of prices or returns of cryptocurrencies as well as of stocks. Among said studies, a wide range of different methods were applied. Many of them point in the same direction however. The overall picture suggests a relationship between social media and prices for both cryptocurrencies and stocks.

## Conclusion

It is clear that this study comes with some restrictions and potentials when one takes the limited scope into consideration. Authors such as Nofer [31] have further developed the idea of sentiment analysis. They have not only taken the social mood on Twitter into account but also the number of followers one has. This attaches more importance to those tweets sent by well-connected users. This, in our eyes, is a better reflection of the reality. It can be assumed that an increased number of followers equals an increased number of people that can be influenced [27]. Although Twitter is one of the biggest social media channels and thus a good indicator for the public mood, there are more signals available that might help predict cryptocurrency returns. It might therefore be valuable to take a bigger variety of data into consideration such as Garcia and Schweitzer [22] have done in their study to predict the return rates of Bitcoins. Returns of other cryptocurrencies or the search volume on Google or Wikipedia are only two possible examples. For this study, data was gathered over a timeframe of 71 days in total. In comparison to most literature, this timeframe is rather short. An extended timeframe would allow for more accurate results. Further potential lies in the applied statistical method. More advanced prediction approaches such as Neural Networks as applied by some authors on cryptocurrencies [18] and many more on stocks [26, 29, 45–50] promise further improvements in the prediction accuracy. The application of Deep Neural Network on the prediction of altcoin returns will be the focus of our future research work. In spite of that, one must acknowledge that we gathered eight samples for 181 altcoins every day. To the best of our knowledge, this is the most detailed analysis of price fluctuations with the most currencies taken into consideration. Among all studied literature we could not find any author who applied a similar approach. Instead, many obtained values once a day. In summary, it can be said that this study applied a different approach in comparison to the existing literature by not focusing on Bitcoins but many different altcoins. Our analysis came up with interesting results that showed a connection between social media activity and sentiment on Twitter and these altcoins. To strengthen the young field of altcoin price predictions, we share the collected data with the community.

## Supporting information

S1 File combined.csv dataset including market data and all input features. The dataset consists of 94,663 samples representing the training and test set. It includes the following columns: Timestamp of query (‘Time’), Cryptocurrency name (‘Cryptocurrency’), Rate (‘Rate’), Trading Volume (‘Volume’), Number of tweets (‘NumTweets’), Mean positive VADER Score (‘Positive’), Mean negative VADER Score (‘Negative’), Mean compound VADER Score (‘Compound’) and Mean neutral VADER Score (‘Neutral’).
(ZIP)
